# Supplementary material for: Developmental nonlinearity drives phenotypic robustness
Source: Nat Commun. 2017 Dec 6;8:1970. doi: 10.1038/s41467-017-02037-7 (PMC5719035; doi:10.1038/s41467-017-02037-7)
Supplement: Supplementary file 1 — Supplementary Information [file 41467_2017_2037_MOESM1_ESM.pdf]

Supplementary Table 1: Pairwise T-test values after FDR multiple testing correction for *Fgf8* mRNA levels.

	Neo/-	+/-	flox/-	flox/-;Crect	flox/+	flox/+;Crect	Neo/Neo	Neo/Wt
+/-	0.0045							
flox/-	0.0392	0.9789						
flox/-;Crect	0.8899	0.0661	0.1285					
flox/+	0.0002	0.0529	0.1614	0.0059				
flox/+;Crect	0.0527	0.4542	0.5993	0.2111	0.0221			
Neo/Neo	0.8899	0.0074	0.0529	0.9789	0.0004	0.0775		
Neo/+	3.98E-05	0.0508	0.2781	0.0064	0.5388	0.0183	8.88E-05	
+/+	1.19E-05	0.0074	0.1434	0.0032	0.8316	0.0045	1.89E-05	0.5388

p < 0.01
p < 0.05
p < 0.1

Supplementary Table 2: P-Values from comparison of morphological disparity.

P0

	+/+	Flox/+	Neo/+	-/+	Flox/-	Flox/ +;Crect	Flox/ -;Crect	Neo/Neo
Flox/+	0.514							
Neo/+	0.908	0.548						
+/-	0.837	0.666	0.873					
Flox/-l	0.749	0.833	0.8	0.875				
Flox/+;Crect	0.596	0.936	0.627	0.702	0.887			
Flox/-;Crect	0.006	0.002	0.003	0.004	0.029	0.002		
Neo/Neo	0.001	0.001	0.001	0.001	0.006	0.001	0.304	
Neo/-	0.001	0.001	0.001	0.001	0.001	0.001	0.001	0.001

p<0.01
p<0.05
p<0.1

E10.5

	+/+	Flox/+	Neo/+	+/-	Flox/-	Flox/-;Crect	Neo/Neo	Flox/-;Crect
Flox/+	0.64							
Neo/+	0.654	0.383						
+/-	0.893	0.724	0.603					
Flox/-	0.769	0.514	0.917	0.689				
Flox/+;Crect	0.801	0.807	0.493	0.916	0.639			
Neo/Neo	0.032	0.025	0.063	0.035	0.086	0.033		
Flox/-;Crect	0.713	0.449	0.955	0.674	0.973	0.579	0.073	
Neo/-	0.001	0.001	0.001	0.001	0.001	0.001	0.001	0.001

p<0.01
p<0.05
p<0.1

Supplementary Table 3: List of genes used in the RNA-seq correlation analysis.

Kegg MapK gene list:

Mapkapk3	Arrb1	Braf	Raf1	Rasa2	Akt1	Akt2	Atf2	Atf4	Bdnf
Cacnb1	Cacnb2	Cacnb3	Cacnb4	Cacng1	Cacng2	Casp3	Cd14	Cdc42	Chuk
Crk	Crkl	Daxx	Ddit3	Dusp2	Egf	Egfr	Elk1	Elk4	Mecom
Cacng5	Fas	Fgf1	Fgf10	Fgf11	Fgf12	Fgf13	Fgf14	Fgf17	Fgf18
Fgf2	Fgf3	Fgf4	Fgf5	Fgf6	Fgf7	Fgf8	Fgf9	Fgfr1	Fgfr2
Fgfr3	Fgfr4	Fos	Gna12	Gng12	Grb2	Hras	Hspa8	Hspb1	Hspa2
Ikbkb	Ikbkg	Jun	Jund	Kras	Stmn1	Rac3	Mapkapk2	Mapkapk5	Max
Mknk1	Mknk2	Mos	Mras	Mapt	Myc	Nf1	Nfatc2	Nfkb1	Nfkb2
Ngf	Nlk	Nras	Ntf3	Ntrk1	Ntrk2	Dusp8	Pak1	Pdgfa	Pdgfb
Pdgfra	Pdgfrb	Prkaca	Prkacb	Prkca	Prkcb	Prkcg	Mapk11	Prkx	Flna
Dusp1	Ptpn5	Ptprr	Rac1	Rac2	Rasgrp2	Rasgrf1	Rasgrf2	Rasgrp1	Rela
Relb	Rras	Sos1	Sos2	Srf	Arrb2	Taok1	Tgfb1	Tgfb2	Tgfb3
Tgfbr1	Tgfbr2	Rasa1	Tnf	Traf2	Traf6	Pak2	Rasgrp4	Dusp7	Akt3
Mapk7	Rasgrp3	Dusp5	Mapk1	Mapk10	Mapk13	Mapk14	Mapk3	Mapk8	Mapk9
Ecsit	Flnb	Mapk12	Dusp4	Ptpn7	Taok3	Taok2	Cacng3	Cacng4	Cacng6
Stk3	Dusp14	Fgf21	Lamtor3	Stk4	Dusp10	Fgf23	Zak	Tab1	Rras2
Fgf22	Dusp6	Tab2	Flnc	Chp2	Dusp16	Dusp3	Nfatc4	Dusp9	Rapgef2
Fgf20	Fgf16	Cacng7	Cacng						

Fgf8 downstream gene list:

Etv5	Fgf8	Fgfr1	Fos	Jun	Etv4	Dusp1	Dusp7	Spry1	Spry2
Fgfr2	Flrt2	Dusp6	Flrt3	Dusp9					

Supplementary Table 4: Gene expression by genotype. Mean of $2^{(Delta-Delta-CT)}$ +/- Standard Deviation of $2^{(Delta-Delta-CT)}$ values.

	Etv4	Fgf17	Fgf4	Prkcg	Rictor	Spry1	Spry4	Trib3
WT	1.08± 0.43	1.06± 0.32	1.46± 1.14	1.12± 0.42	1.1± 0.38	1.02± 0.22	1.02± 0.22	1.11± 0.63
Neo/ +	0.93± 0.33	1.23± 0.17	0.78± 0.51	1.08± 0.41	1.11± 0.35	0.84± 0.10	1.04± 0.17	1.19± 0.19
WT/-	1.06± 0.35	0.96± 0.24	2.29± 1.51	1.19± 0.40	1.2± 0.28	0.86± 0.16	0.89± 0.17	1.08± 0.93
Neo/Neo	0.56± 0.40	0.49± 0.20	3.93± 2.82	0.65± 0.40	0.98± 0.16	0.81± 0.25	0.64± 0.03	3.99± 2.60
Neo/-	0.56± 0.15	0.12± 0.06	2.27± 1.09	0.8± 0.61	1.11± 0.54	0.61± 0.39	0.64± 0.26	3.11± 1.97

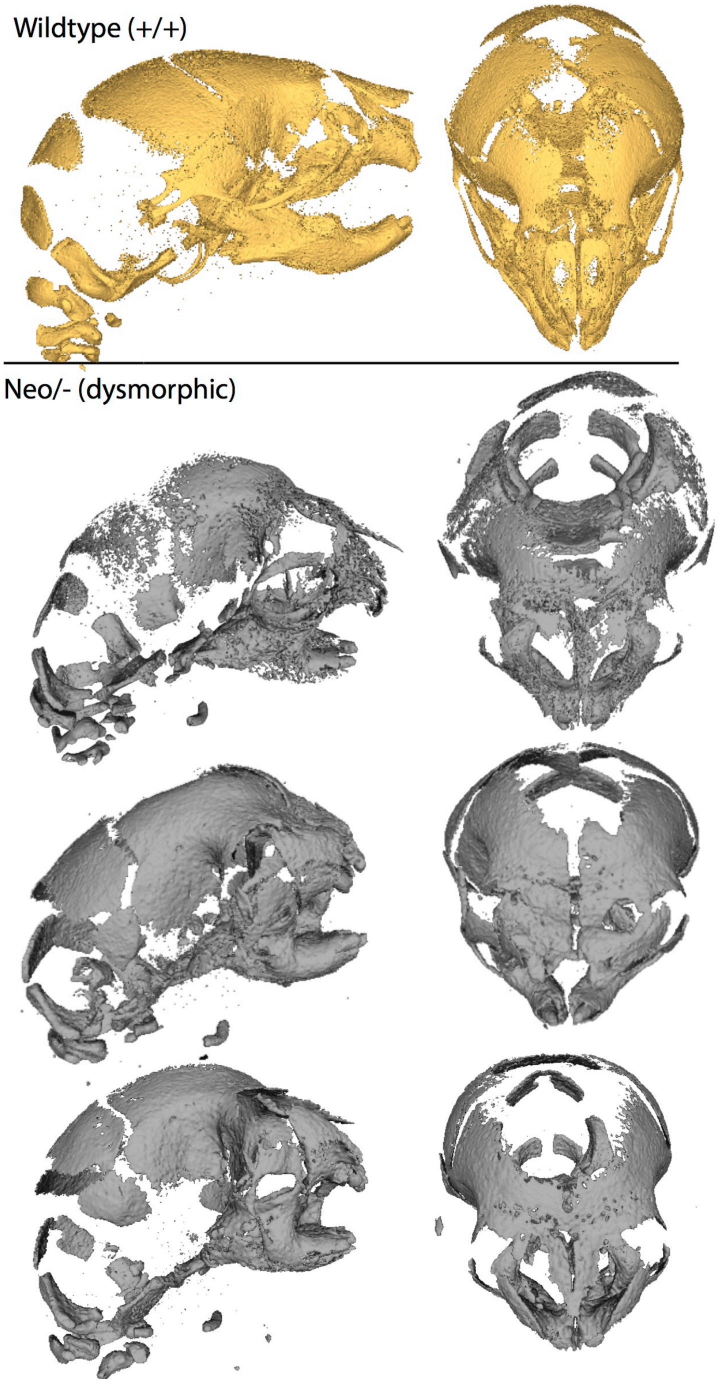

Supplementary Figure 1: Dysmorphic skulls in *Fgf8* Neo/- lack identifiable landmarks. Top: Wildtype P0 embryo showing side (left) and facial (right) views. Bottom: 3 P0 *Fgf8* Neo/- (mutant) embryos. All three neonate have shortened mandibles. The top skull shows missing cranial bone, and skeletal bending as well as alterations in the orbital region and the zygomatic arch. The middle embryo shows a midline cleft, general dysmorphology in the parietal and frontal regions as well as a loss of the left orbital. The bottom embryo shows loss of parts of the zygomatic arch and frontal bones as well as an overall shortening of the frontal bone.

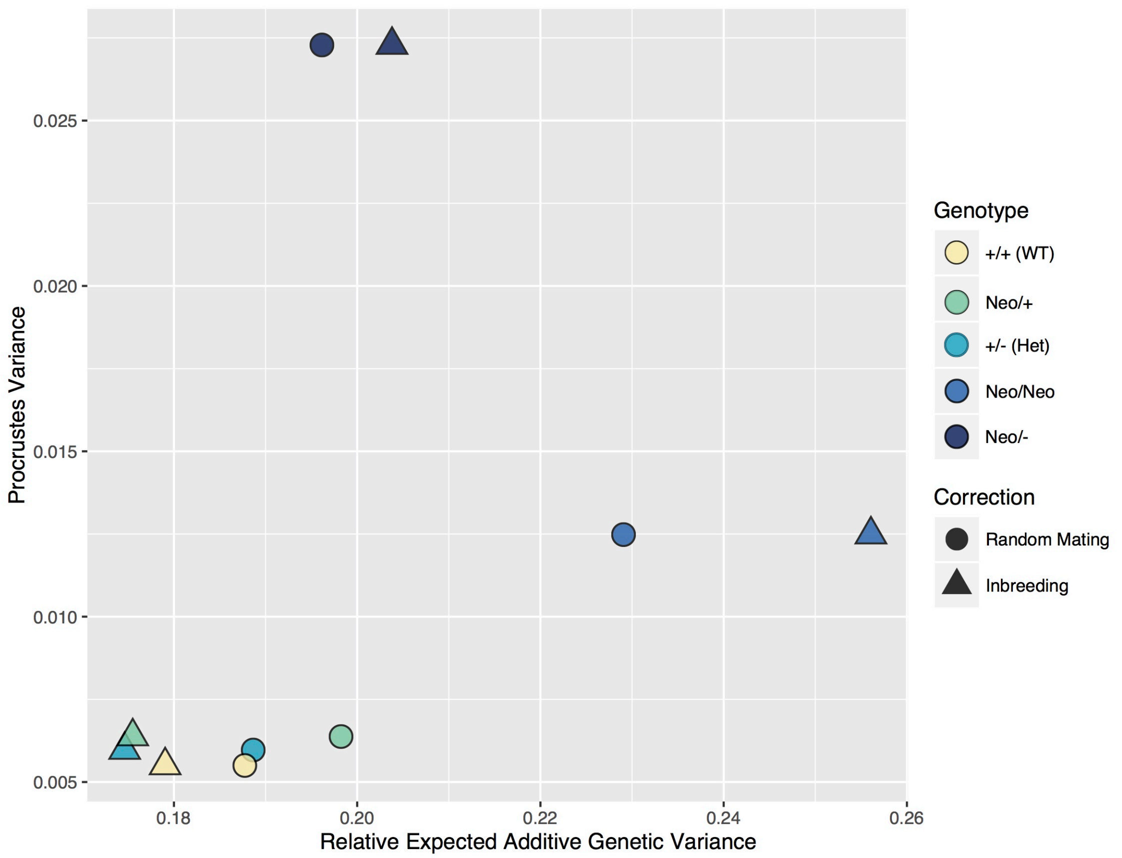

Supplementary Figure 2: Genetic Variance vs Phenotypic Variance. The additive genetic variance either corrected for inbreeding or assuming random mating from the SNP analysis was compared to the Procrustes variance from the shape data to confirm that there is no detectable relationship between genetic variance within the genotypes and Procrustes variance. Correlation between the random mating additive variance and procrustes variance =0.18, p-value=0.77, 95%CI = -0.83 - 0.91. Correlation between the inbreeding corrected additive variance and procrustes variance =0.39, p-value=0.51, 95%CI = -0.74 - 0.94.
